# Enhancement of Physical Properties and Corrosion Resistance of Al-Cu-Al_2_O_3_/Graphene Nanocomposites by Powder Metallurgy Technique

**DOI:** 10.3390/ma15207116

**Published:** 2022-10-13

**Authors:** Omayma A. El-Kady, Hossam M. Yehia, Fathei Nouh, Ibrahim M. Ghayad, Taher El-Bitar, Walid M. Daoush

**Affiliations:** 1 Powder Technology Department, Manufacturing Technology Institute, CMRDI, Cairo 11913, Egypt; 2 Production Technology Department, Faculty of Technology and Education, Helwan University, P.O. Box 11795, Cairo 11281, Egypt; 3 Mechanical Department, Faculty of Engineering, Sinai University, Arish 45511, Egypt; 4 Plastic Deformation Department, Metals Technology Institute, CMRDI, Cairo 11913, Egypt; 5 Department of Chemistry, College of Science, Imam Mohammad Ibn Saud Islamic University (IMSIU), P.O. Box 90950, Riyadh 11623, Saudi Arabia

**Keywords:** powder metallurgy, aluminum, graphene nanosheets, electrical conductivity, corrosion rate, thermal conductivity, thermal expansion

## Abstract

In this study, we enhanced the adhesion of graphene nanosheets to achieve homogeneous dispersion, consequently improving the electrical and thermal conductivity, coefficient of thermal expansion, and corrosion resistance with an aluminum matrix containing up to 1.5 wt. % graphene. First, 2.5 wt. % Al_2_O_3_ and varying ratios of graphene up to 1.5 wt. % were coated with 5 wt. % silver nanoparticles to metalize their surfaces. Predetermined portions of coated alumina and graphene were mixed with Al/10 wt. % Cu powder for 45 h. Mixed samples were compacted under 600 MPa and sintered at 565 °C in a vacuum furnace for 60 min with a low heating rate of 2 °C/min. The strengthening effect of the added materials on the density, microstructure, electrical and thermal conductivities, thermal expansion, and corrosion behavior of aluminum were investigated. Excellent adhesion and homogeneous dispersion of the investigated reinforcements were achieved. Three phenomena were observed: (1) an improvement in the densification, electrical and thermal conductivity, thermal expansion, and corrosion rate by adding 10 wt. % Cu to the aluminum matrix; (2) deterioration of the properties of Al/10 wt. % Cu with the addition of 2.5 wt. % alumina nanoparticles; and (3) improved properties with the addition of graphene nanosheets up to 1 wt. % and a decrease in property values beyond 1.5 wt. % graphene content due to the formation of agglomerations and pores in the metal matrix.

## 1. Introduction

Composites, a necessary new class of materials that differ from alloys, can be obtained by combining one or more reinforcement-dispersed phases with varying physical, mechanical, and chemical properties in a matrix phase. They can be categorized into three primary types according to the matrix phase, namely polymer matrix composites (PMCs), metal matrix composites (MMCs), and ceramic matrix composites (CMCs). Alloys and composite materials with high physical and mechanical properties, high specific density, and high resistance to chemical corrosion, especially in coastal cities, are required for many industrial applications. The corrosion characteristic provides essential information with respect to products used in heat exchangers construction, architecture, transportation, and many other applications [1,2,3,4,5,6,7]. Aluminum is a unique material characterized by its low density and thermal properties. However, it suffers from low corrosion resistance in corrosive environments, such as seawater. Traditional methods used to fabricate aluminum–copper–graphene alloys require a process temperature higher than 800 °C; in addition, a thin layer of aluminum carbide (Al_4_C_3_) can be formed during the manufacturing process at this temperature, as detected by X-ray analysis [8,9,10]. The formed aluminum carbide layers damage aluminum–graphene alloys in corrosive environments. The irregular pitting caused by stress corrosion leads to cracks, which are highly sensitive to chemicals. This limits the widespread use of these alloys, especially in coastal cities [9]. Aluminum carbide is formed when metal-containing graphene is cast at a temperature higher than 800 °C [10]. Therefore, the powder metallurgy technique can be used instead of casting to produce aluminum–copper–graphene composites. Aluminum composites are fabricated at a temperature lower than their melting temperature to avoid the formation of aluminum carbide and to improve its chemical corrosion resistance. Aluminum–copper alloy is protected as a result of many factors, such as temperature, humidity, and wind-carrying chloride ions, which further enhance and accelerate the corrosion process [11].

Alloying or reinforcement of aluminum with different types of alloying elements and reinforcing particles, as well as heat treatment, are considered the most critical solutions to improve the strength and increase the corrosion resistance of aluminum [1,2,3,4,5,6]. Elkady et al. studied the influence of 0.2 wt. % GNs (graphene nano sheets) and varying ratios of SiC up to 25 wt. % on the hardness, wear resistance, and corrosion rate of aluminum matrices [6]. The addition of 0.2 wt. % GNs to an aluminum matrix was found to increase the hardness value by 47.38%, and by reinforcement with 20 wt. % SiC increased the hardness by 56.29%. The wear rate gradually decreased to 15 wt. % and then increased with the addition of 20 wt. % SiC. Reinforcing the Al-Ni-GNs with 10 wt. % SiC led to a reduction in substrate resistance toward the electrochemical deposition process and consequently improved the electrodeposition of ZnO film up to 20 wt. % SiC [6].

Aluminum and its alloys are a class of material with excellent corrosion resistance, even in aqueous media. However, in many cases, they cannot withstand attacks by reactive chloride ions [12]. Corrosion increases due to the penetration and diffusion of chloride ions and their accumulation on the aluminum oxide layer, which covers and protects the aluminum surface, leading to the deterioration of its protective property. Several researchers have developed protection systems for aluminum–copper alloys, effectively preventing metal corrosion. Aluminum–copper alloys can be protected by chrome plating [13]. However, chromium compounds are highly toxic to the environment and expensive. Research on ecofriendly materials for anticorrosion systems for alloys is still limited. Graphene is an excellent alternative material to protect aluminum–copper alloys from corrosion [14]. Graphene was used as a coating material for the entire surface of aluminum alloys, providing effective corrosion resistance in multiple media [15]. It also has many advantages making it suitable for use in several engineering applications, such as high surface area and chemical inertness. It shows a high corrosion resistance, making it a candidate for corrosion protection. Flat graphene is characterized by gaseous oxygen impermeability, which supports its anticorrosion nature. The corrosion resistance of graphene as an excellent coating material was studied by Li et al. [16], who investigated the effect of homogeneously distributed graphene nanoplatelets (GNPs) as a long-term corrosion inhibitor on aluminum corrosion behavior. The deformation-driven metallurgy technique was applied to prepare Al/1.5 wt. % GNP composites at varying rotational speeds. The process effectively enhanced the grain refinement and dispersion of GNPs via severe plastic deformation. Increased corrosion resistance was achieved as a result of the high homogenous distribution and the formation of a protective oxide film [17,18].

Alumina particles, a common ceramic oxide among metal oxides, can be used to reinforce the aluminum matrix of metal matrix composites. They are cost-effective, with stable properties, even at high temperatures [19]. The hot-powder method was used to achieve good adhesion and full densification and, consequently, improve the deficiency of mechanical properties in an aluminum matrix [7]. The aluminum matrix was reinforced with varying amounts of alumina up to 25 wt. %. The density of the alumina did not influence the density of the Al-10Ni-10Cu. Owing to the high stiffness of the alumina and its ability to form intermetallics, the hardness and wear rate were improved, and the coefficient of thermal expansion was enhanced. The influence of hybrid (Al_2_O_3_-GNs) on the properties of the Al-5Ni-0.5Mg matrix was studied [20]. The results revealed that the addition of 7.5 wt. % of this new combined reinforcement increased the strength and electrical properties of the aluminum matrix, and the electrical conductivity increased from 28.6 MS/m to 66.7 MS/m. Hossam M. H. et al. [21] studied the effect of 10 wt. % Al_2_O_3_ and 10 wt. % Al_2_O_3_ /10MoS_2_ on the density, hardness, and wear rate of copper. The hardness and wear rate decreased significantly despite a decrease in the density with the addition of alumina.

This aim of the present study is to manufacture a new generation of a material composed of Al-Cu-Al_2_O_3_/x GNs by powder technique that possesses high corrosion resistance and thermal expansion coefficient and is suitable for both electrical and electronic applications.

## 2. Materials and Methods

### 2.1. Materials

**Figure 1:**
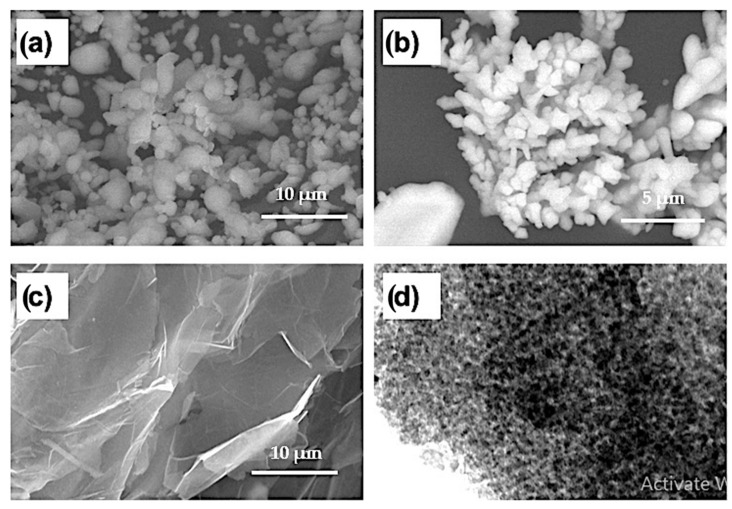
(**a**–**c**) SEM images of Al powder, Cu powder, and graphene nanosheets and (**d**) TEM image of Al_2_O_3_ nanoparticles.

Aluminum powder with a particle size of 2–3 µm and a purity of 99.9% was purchased from Loba Chemie Pvt. Ltd., Mumbai, India. Copper powder with a dendritic shape, a particle size of 3–5 µm, and a purity of 99.5% was supplied by DOP ORGANİK KİMYA SAN.VE TİC. LTD ŞTİ (İstanbul, Turkey). Alumina nanoparticles with a particle size of 50–100 nm (99.9% purity) and 2–10 nm thick two-dimensional graphene nanosheets (99.9% purity) were purchased from Hart Minerals (Wakefield, MA, USA) and ACS Material, LLC (Medford, MA, USA), (an advanced chemical supplier), respectively. Figure 1 shows electron microscope images of the investigated powders.

### 2.2. Methods

#### 2.2.1. Fabrication of Sintered Nanocomposites

**Figure 2:**
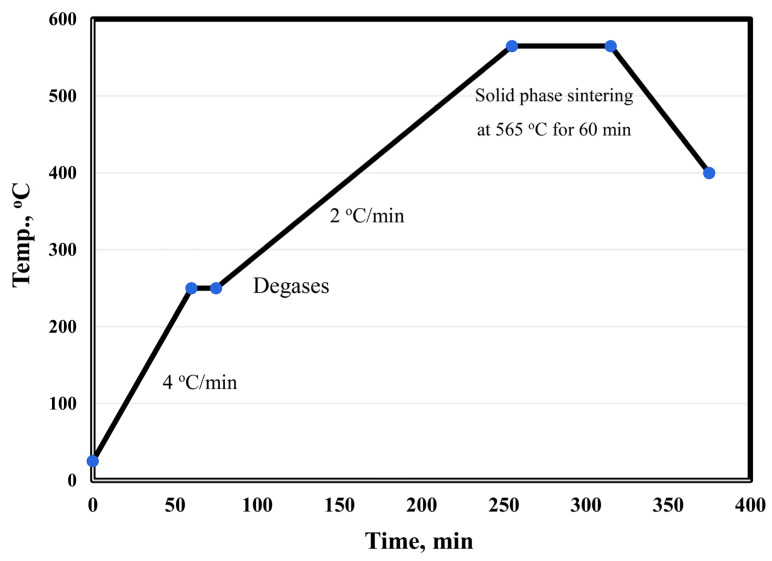
Heating cycle curve of the Al-Cu-Al_2_O_3_/GNs nanocomposite fabrication process.

The surface of alumina and graphene nanosheet reinforcements was chemically treated to remove contamination by stirring in 10 wt. % NaOH and acetone for 1 h, respectively. Then, the treated reinforcements were coated with 5 wt. % nano-Ag by the electroless plating process [20,21,22,23,24,25]. Silver nitrate was dissolved (3 gm) in 1 L of distilled water, and the pH of the solution was adjusted to 11; then, the reinforcement powder was added. Stirring was continued for 5 min to ensure the dispersion of the powder particles in the solution. Formaldehyde solution (38%) was added to 85 vol. % of the used water. The reaction was completed within 30 min. The powders were filtered and then dried at 80 °C in a drier for 3 h. The main objective of the metallization process of graphene and Al_2_O_3_ was to improve their wettability and adhesion with aluminum matrix during the sintering process. Six samples were prepared: Al, Al-10Cu, Al-10Cu/2.5Al_2_O_3_, Al-10Cu-2.5Al_2_O_3_/0.5GNs, Al-10Cu-2.5Al_2_O_3_/1GNs, and Al-10 Cu-2.5 Al_2_O_3_/1.5GNs. After adjusting the composition of the samples, they were mechanically alloy-milled (MAM) in a stainless-steel container for 45 h to increase the homogeneous distribution between all of the sample constituents. Liquid hexane (5%) was used during the mixing process as a processing agent to prevent the accumulation of nanoparticles, which may be produced during the milling process. The process was performed with a 1:10 powder-to-ball ratio at 350 rpm. Then, the samples were compacted with a uniaxial press into a 10-mm diameter die at 600 MPa. The diameter of the used alumina balls was 12 mm. The samples were sintered at 565 °C in a vacuum furnace for 60 min with a low heating rate of 2 °C/min. Figure 2 shows the sintering cycle of the fabrication process.

#### 2.2.2. Characterization of the Sintered Nanocomposites

The produced sintered samples were characterized by studying their density, microstructure, electrical conductivity, thermal conductivity, coefficient of thermal expansion, and chemical corrosion behavior. The density of the sintered samples was evaluated by Archimedes’ method according to the MPIF 42, 1998 standard. A Quanta FEG 250 scanning electron microscope (SEM) (FEI Company, Dresden, Germany) equipped with EDAX analysis was used to investigate the microstructure of the sintered sample. The electrical conductivity of the fabricated nanocomposite was measured at 25 °C and 60 kHz with a PCE-COM 20 tester (PCE Instruments, Meschede, Germany). The thermal conductivity was evaluated based on the electrical conductivity results according to the Wiedemann–Franz Equation (1) [26].

(1) $$\frac{\lambda }{\sigma T}=\frac{\pi^{2}k_{B}^{2}}{3e^{2}}=L=2.443\times 10^{-8\;}w/K^{2}$$

where *λ* is thermal conductivity (W/mK), *σ* is electrical conductivity (Ω m^−1^), *T* is absolute temperature (K), *k_B_* is the Boltzmann constant, and *L* is the Lorentz number. The thermal strain was used to determine the samples’ thermal expansion coefficient (CTE) according to Equation (2) [7]. The thermal strain was evaluated at different temperatures from 50 °C to 350 °C in 50 °C increments for 30 min. The height of each sample before and after heating was measured using a digital indicator with 0.0001 mm accuracy.

(2) $$\alpha =\frac{\varepsilon }{∆T}{\;\text{°}C}^{-1}$$

where α is the thermal expansion (°C^−1^), Δ*T* is the temperature difference between the heating temperature and room temperature (°C), and ε is the thermal strain (h_2_-h_1_).

An electrochemical test was performed to study the corrosion behavior of the fabricated samples using an AUTO LAB PGSTA30 working station (Utrecht, The Netherlands) at room temperature. A 3.5 wt. % NaCl electrolyte composition solution was used to perform the test. Samples with a 0.204 cm^2^ surface area were polished using 1200 SiC paper. A platinum counter electrode and a saturated reference calomel electrode (SCE) were used, with the sample used as a working electrode. In each sample’s open-circuit potential (OCP), the current was measured between the counter and working electrode, whereas the potential was measured between the reference and working electrodes. Each sample was continuously monitored for 30 min in the electrolyte. Then, a potentiodynamic polarization test was performed up to 3.0 V with a 2 mV s^−1^ scan rate. Measurements were carried out at ambient temperature under aerated conditions. Each sample test was repeated two times. Tafel analysis estimated the corrosion potential (*E*_corr_) and corrosion current density (*I*_corr_) based on the polarization plots.

## 3. Results and Discussion

### 3.1. Density Measurements

**Figure 3:**
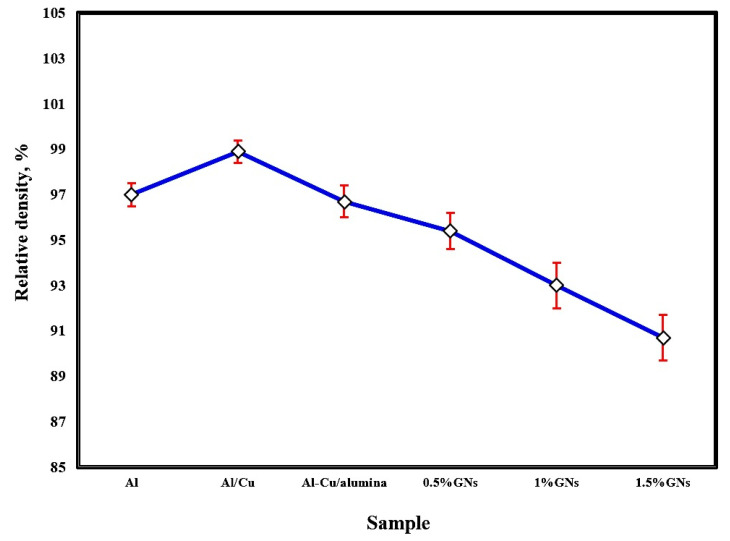
Relative density of the fabricated sintered samples at 565 °C.

The effect of sintering temperature and the addition of copper, alumina nanoparticles, and varying percentages of graphene nanosheets on the relative density of aluminum is represented in Figure 3. The sintering process at 565 °C achieved a satisfactory relative density. Improving the density by increasing the sintering temperature enhanced the adhesion between the particles, consequently reducing porosity. The addition of 10 wt. % Cu to the Al matrix increased the total composite density due to the high density of copper 8.9 g/cm^3^ and its excellent distribution and adhesion with the Al matrix, enhancing the wettability between them. By adding 2.5 wt. % Al_2_O_3_ nanoparticles to the Al-10 wt. % Cu sample, the density was decreased due to the low density of alumina (3.95 g/cm^3^). Furthermore, the density of Al-10Cu-2.5Al_2_O_3_ was gradually reduced by increasing the graphene nanosheet content, possibly as a result of the density of graphene nanosheets being lower than that of both metallic Al and Cu (about 2.2 g/cm^3^) or because with increased graphene nanosheet content, agglomerations formed, decreasing the wettability with the aluminum matrix and enhancing the formation of pores [27,28].

### 3.2. Microstructure of Sintered Nanocomposites

**Figure 4:**
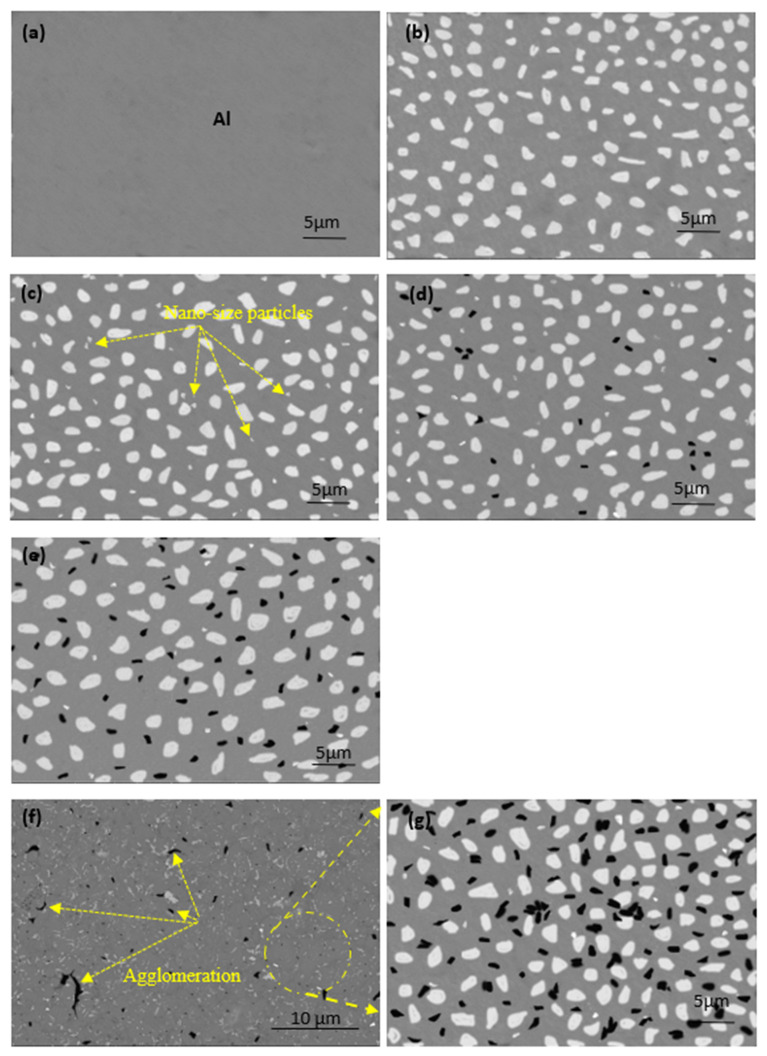
SEM images (BSE mode) of the microstructure of the fabricated samples at 565 °C: (**a**) pure aluminum, (**b**) Al/10Cu, (**c**) Al-10Cu/2.5 nano-Al_2_O_3_, (**d**) Al-10Cu-2.5Al_2_O_3_/0.5 GNs, (**e**) Al-10Cu-2.5Al_2_O_3_/1 GNs, and (**f**,**g**) Al-10Cu-2.5Al_2_O_3_/1.5 GNs.

The microstructures of the produced samples fabricated at 565 °C are shown in Figure 4. An ideal microstructure for all samples was achieved under the processing conditions described above. Four different regions are observed: the gray region, representing the Al matrix, and the large white spots indicating to copper metal. Nano-alumina was not evident in image 3, appearing as tiny white spots and starting to appear in the microstructure of samples (c to f), which contain Al-10Cu-2.5Al_2_O_3_. The fourth region is the black spots in the image (d), representing the GNs that appeared with increased content. Because the sintering process was performed at a low heating rate, excellent cohesion was established at the interface of the grain boundaries between all ingredients, and no pores were formed. In image c, alumina nanoparticles are observed in the region between the copper particles. The reinforced copper particles are represented by an irregular shape, regardless of the excellent distribution of graphene nanosheets with the Al matrix at all GN concentrations. Accumulations were observed in the samples containing 1.5 wt. % GNs, as shown in images (f and g). This is due to the high graphene ratio, in which 5 wt. % GNs coated with silver nanoparticles was insufficient for complete capsulation of 1.5 wt. % GNs; therefore, this is the only sample exhibiting such behavior due to the non-wettability problem with the Al matrix [29]. This accumulation may be the result of an insufficient amount of liquid hexane processing agent.

**Figure 5:**
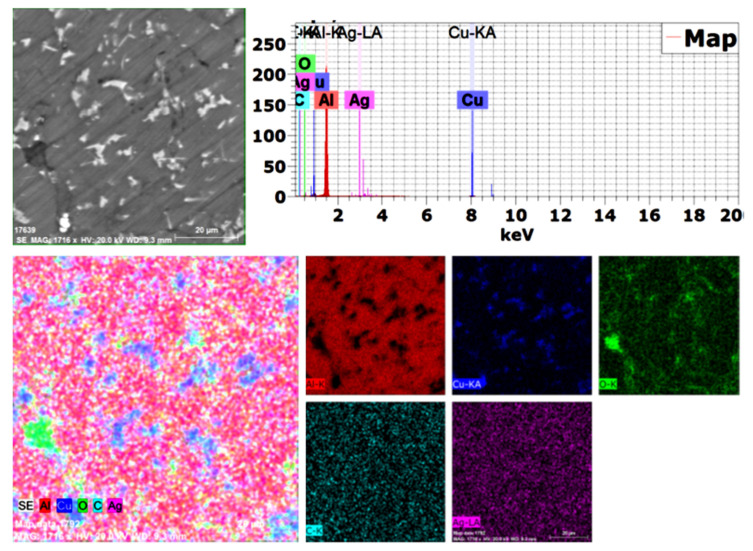
Mapping analysis of the produced 1.5 wt. % GNs sintered sample.

Because the used alumina particles were nano-sized and not detected by SEM (BSE), mapping was performed with EDAX analysis, as shown in Figure 5. All used elements were confirmed, and no impurities or foreign elements were detected, with excellent distribution of all elements.

### 3.3. Electrical Conductivity of Sintered Nanocomposites

**Figure 6:**
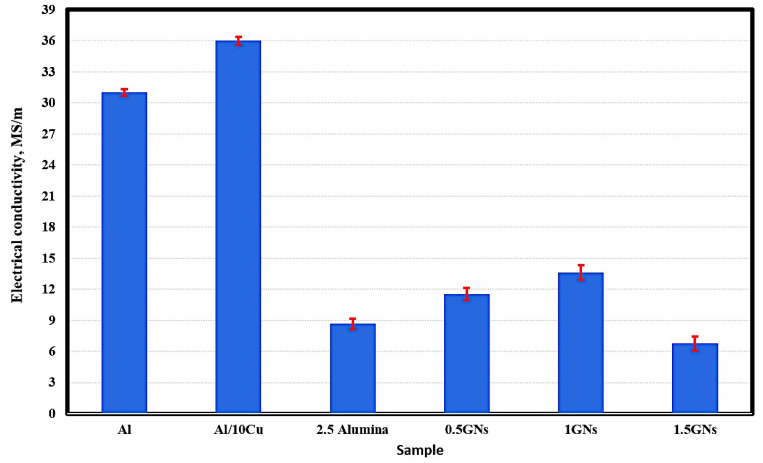
Electrical conductivity of the produced sintered nanocomposites.

Figure 6 represents the electrical conductivity behavior of the fabricated samples; the results show that the electrical conductivity of pure aluminum reached 31 MS/m, and by adding 10 wt. % Cu, it was improved to 36 MS/m. This increment is due to the high conductivity of copper metal (58.7 MS/m) relative to that of aluminum metal (36.9 MS/m). Additionally, the optimum conditions under which the samples were produced may have improve the bonding between aluminum and copper grains, decreasing air gaps in the area of the grain boundaries, facilitating the movement of electrons between them, and decreasing the resistance of the electric current [30].

By adding 2.5% alumina nanoparticles, the electrical conductivity of aluminum was decreased significantly from 36 MS/m to 8.66 MS/m due to the poor electrical conductivity of the alumina nanoparticles, which are classified as an insulator that resists the motion of electrical charge carriers [31].

The effect of graphene nanosheets was observed; their addition to the Al-10Cu-2.5Al_2_O_3_ nanocomposite increased the electrical conductivity from 8.66 MS/m to 11.54 MS/m at 0.5 wt. % GNs with 33.25 wt. % increments. By increasing the graphene nanosheet content to 1 wt. %, the electrical conductivity was increased to 13.62 MS/m, then decreased to 1.5 wt. % and reaching 6.67 MS/m. The decrease in the electrical conductivity at 1.5 wt. % GNs is due to the increase in GN agglomeration. The percentage of graphene agglomerates naturally increases the rate of interstitial spaces that resist the movement of electrons, reducing the passage of electric current [31].

### 3.4. Thermal Conductivity of Sintered Nanocomposites

**Figure 7:**
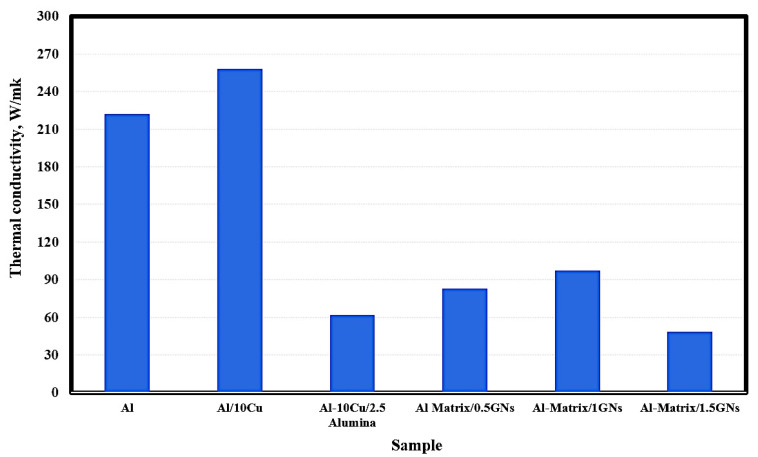
Thermal conductivity of the fabricated sintered nanocomposites.

The thermal conductivity of the fabricated samples is shown in Figure 7. The pure metallic aluminum exhibited thermal conductivity of 222 W/mK, and by adding 10 wt. % Cu, this value was increased to 258 W/mk. The addition of 2.5 wt. % Al_2_O_3_ nanoparticles reduced the thermal conductivity of Al/10 Cu from 258 to 62 W/mK. The observed reduction in the thermal conductivity of Al/10 Cu may be due to the low thermal conductivity of alumina nanoparticles, which are classified as a non-conductive ceramic material. Because graphene has a high thermal conductivity of 4000 W/mK, that of Al/10 Cu/2.5 Al_2_O_3_ was increased from 62 to 83 and 97.5 W/mK at 1 wt. % GNs, then decreasing to 48.4 W/mK for 1.5 GNs samples. The reduction in thermal conductivity in the case of the 1.5 wt. % GNs sample may be due to the formation of agglomerations in the metal matrix, which increase the air gaps between the reinforcements and the matrix and decrease the conduction behavior of the composite material. In other words, accumulation leads to the formation of micropores and air gaps that restrict the movement of electrons and reduce the thermal conductivity [32].

### 3.5. Coefficient of Thermal Expansion of Sintered Nanocomposites

Reinforcing the Al matrix by material with low CTE with homogeneous distribution helps to decrease the CTE of the formed composite because all the dispersed reinforcements act as an internal network that restricts and hinders the expansion process [32].

**Figure 8:**
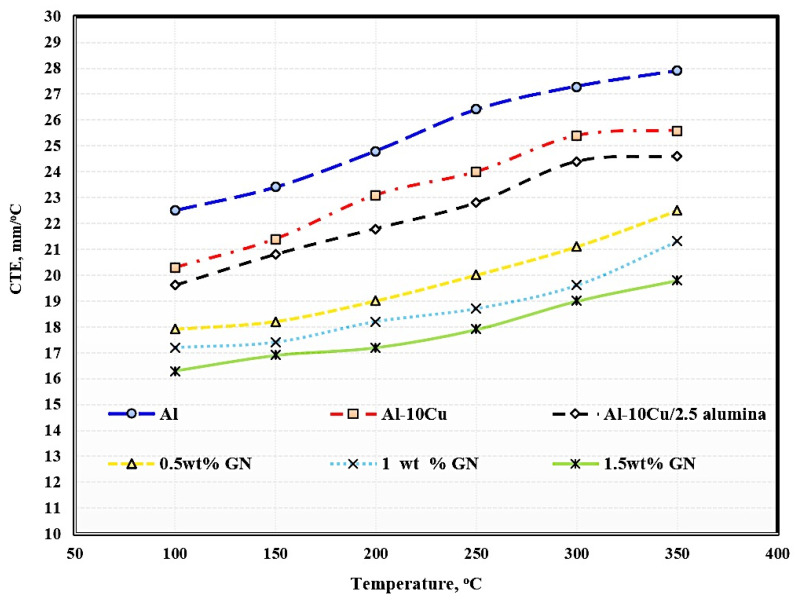
Coefficient of thermal expansion of the produced sintered samples.

The effects of temperature, the addition of 2.5 wt. % Al_2_O_3_ nanoparticles, and the addition of varying graphene nanosheet contents on the CTE of aluminum are shown in Figure 8. Regarding the temperature impact, it is clear that the higher the operating temperature, the greater the thermal expansion. The increase in thermal expansion is due to the increase in the kinetic energy of atoms as a result of increased temperature. Temperature is an essential function of kinetic energy responsible for the movements of atoms and molecules composed of the entire material structure. When any material is exposed to temperature, the atoms and molecules absorb heat, which is converted to heat energy, increasing the vibrations of the atoms and molecules; by increasing the temperature, the movement of the atoms and molecules in increased, and the distance between them also increases, leading to increased sample dimensions.

With the addition of copper, alumina nanoparticles, and graphene nanosheets, it is clear that the thermal expansion of aluminum was decreased because the thermal expansion of copper, alumina, and graphene is lower than that of aluminum. Another factor that led to the decrease in thermal expansion is the growth of graphene agglomerates with increased graphene, leading to the formation of pores among the agglomerated chips and a consequent decrease in thermal expansion. Pure aluminum exhibited a thermal expansion of 5.4, whereas the samples reinforced with Cu, Al_2_O_3_ nanoparticles, and varying wt. % GN contents exhibited thermal expansion of 5.3, 5, 4.6, 4.1, and 3.5, respectively.

### 3.6. Corrosion Behavior of Sintered Nanocomposites

**Figure 9:**
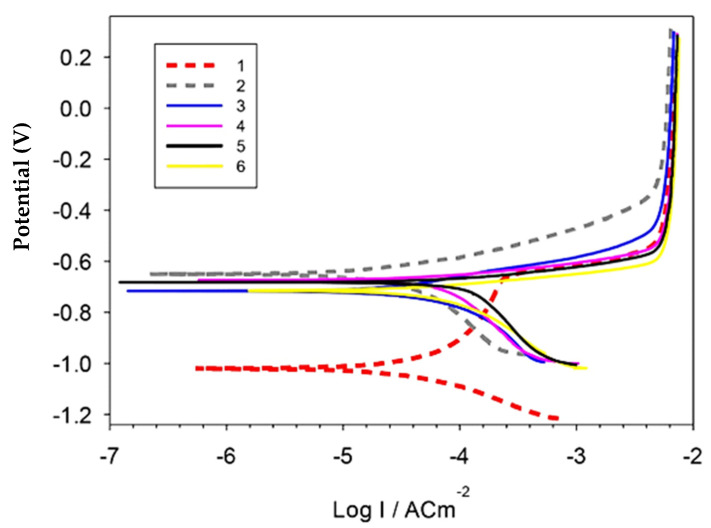
Potentiodynamic polarization curves of the investigated sintered samples in 3.5% NaCl solution.

Figure 9 and Table 1 represent the polarization curves and corrosion parameters of the produced Al, Al-10Cu, Al-Cu/2.5 nano-Al_2_O_3_, Al-Cu-nano-Al_2_O_3_/0.5 GNs, Al-Cu-nano-Al_2_O_3_/1 GNs, and Al-Cu-nano-Al_2_O_3_/1.5 GNs nanocomposites in 3.5 NaCl solution. The polarization behavior of the Al matrix was changed by adding copper, alumina nanoparticles, and varying percentages of graphene nanosheets. The addition of copper nanoparticles shifted the E_corr_ in the negative direction by about −0.4 V and decreased the corrosion rate from ≈0.78 to ≈0.58 mm/yr. The Al-10Cu sample also showed a pseudo-passive region. The addition of 2.5% alumina nanoparticles to the Al-10Cu primarily increased the corrosion rate to 1.449 mm/yr, which is the highest value among all samples. The addition of alumina nanoparticles may increase the boundaries of the particles exposed to the solution, thus increasing the rate of chemical corrosion. The addition of 0.5–1 wt. % graphene nanosheets to the Al-10Cu-2.5Al_2_O_3_ decreased the corrosion rate to 1.27 and 0.98 mm/yr, respectively. Further addition of graphene nanosheets resulted in an increase in the corrosion rate. The significant reduction in the corrosion rate for the Al/10Cu sample can be attributed to the enhancement of the corrosion resistance of Al as a result of the addition of more noble Cu metal and the disappearance of pores in the microstructure. On the contrary, the increase in corrosion rate in the presence of Al_2_O_3_ nanoparticles can be attributed to the presence of pores in the Al-Cu-Al_2_O_3_ matrix, which facilitated the penetration of the corrosive medium into the sample, causing its corrosion. However, the addition of GNs significantly improved the corrosion resistance of the Al-Cu-Al_2_O_3_ nanocomposite. GNs improve the Al-Cu-Al_2_O_3_ nanocomposite surface quality as a result of their inertness and distribution in the Al matrix. Furthermore, GNs exhibit good corrosion resistance, so reinforcing an Al matrix GNs decreased the corrosion rate and protected the investigated samples from the corrosive solution [33]. The graphene layer has a large specific surface area relative to its volume ratio, and its addition to the Al-Cu-Al_2_O_3_ nanocomposite resulted in thorough distributed on the interface of a large number of grain boundaries that may be subject to corrosion, leading to a reduction in the rate of chemical corrosion. The corrosion rate for the 1.5 wt. % GN sample was high due to the formation of pores in the GN agglomeration regions, facilitating the penetration of the corrosive medium into the composite. Additionally, the increase in the exfoliated graphene nanolayers activates the galvanic corrosion of Al, increasing the corrosion rate [20].

## 4. Conclusions

Based on the obtained results, the following conclusions can be drawn:

1. Aluminum matrix nanocomposites containing high content of graphene nanosheets up to 1.5 wt. % were successfully produced by powder metallurgy at a temperature of 565 °C and a low heating rate of 2 °C/min;
2. An excellent distribution of all integrates was achieved as a result of performing the mixing process in the presence of liquid hexane as processing agent for 45 h;
3. Using a slow heating rate of 2 °C/min provided an opportunity for sufficient bonding between the grain boundaries, as well as the elimination of pores and air gaps;
4. Some accumulations of graphene flakes appeared in the sample containing 1.5 wt. % GNs, which can be avoided by increasing the percentage of liquid hexane;
5. Mapping analysis emphasized the chemical composition and distribution of all elements;
6. The electrical conductivity and thermal conductivity of Al were improved by adding copper and deteriorated by adding alumina nanoparticles due to its poor electrical conductivity as a non-conductive material. However, adding graphene nanosheets improved the electrical and thermal conductivity of Al;
7. The 1.5 wt. % GNs sample recorded 16.3 m/°C at 50 °C and 19.8 mm/°C at 350 °C, with a thermal expansion range of 3.5; and
8. The corrosion rate was decreased from 0.74 mm/year for aluminum to 0.57 mm/year for Al/10Cu. The addition of 2.5 wt. % Al_2_O_3_ increased to 1.44 mm/year, and with the addition of GNs, it was decreased to 0.97 mm/year at 1 wt. % GNs and increased at 1.5 wt. % GNs due to the accumulation of GNs.

## Figures and Tables

**Table 1:** Corrosion parameters of Al/(Cu-GNs) nanocomposites obtained from electrochemical measurements in 3.5% NaCl solution.

Corrosion Rate (mm/year)	Ba (V/dec)	Bc (V/dec)	I_corr_ (A/cm^2^)	E_corr_ (V/SCE)	Materials
0.74873	0.48373	0.17274	6.6879 × 10^−5^	−1.0216	Pure Al
0.57565	0.1168	0.42918	5.0313 × 10^−5^	−0.65102	Al/10Cu
1.4495	0.121	0.372	0.000109	−0.717	Al-Cu 2.5%Al_2_O_3_
1.2704	0.062	0.439	0.0001	−0.675	Al-Cu-Al_2_O_3_/0.5GNs
0.9784	0.06	0.409	8.08 × 10^−5^	−0.7122	Al-Cu-Al_2_O_3_/1GNs
1.4024	0.055	0.337	0.00011	−0.716	Al-Cu-Al_2_O_3_/1.5GNs

## Data Availability

All the data is available within the manuscript.
